# Supplementary material for: Genome-wide association mapping identifies yellow rust resistance loci in Ethiopian durum wheat germplasm
Source: PLoS One. 2021 May 17;16(5):e0243675. doi: 10.1371/journal.pone.0243675 (PMC8128278; doi:10.1371/journal.pone.0243675)
Supplement: Attachment — Submitted filename: Response to Reviewers.docx [file pone.0243675.s006.docx]

**Date: 06-Apr-21**

**To Academic Editor,**

**PLOS ONE**

Subject: **Response to Reviewers**

**Dear Dr. Aimin Zhang,**

I am so grateful for the time allowed to me to work on the points raised by you (the Academic Editor) and both Reviewer’s. All the points are very relevant, clear and constructive and I am thankful for that again.

I have now finished working on it exerting as much effort as I could. I have given a sort of reflection on the answers the reviewers gave to the points raised by the Academic Editor while answers to questions raised by the reviewers and amendments wherever they are suggested. To make reading easier, I have used a red font colour for the reactions I gave while keeping the questions as they are, but green highlight for the numbers of the comments and questions. Wherever it is relevant, I have indicated the Line numbers of the texts where the changes have been made in the revised manuscript with the Track Changes. I put all my reaction to the points raised sequentially to both reviewers as they appeared in the revision email text originally received. Just in case two separate files are needed per reviewer, I am ready to provide that.

I hope I have addressed all the points and look forward getting your reply soon.

Best Regards,

Sisay Kidane Alemu

**My Reactions to all the points raised by the Academic Editor and Reviewers**

Reviewers' comments:

Reviewer's Responses to Questions

**Comments to the Author**

1. Is the manuscript technically sound, and do the data support the conclusions?

The manuscript must describe a technically sound piece of scientific research with data that supports the conclusions. Experiments must have been conducted rigorously, with appropriate controls, replication, and sample sizes. The conclusions must be drawn appropriately based on the data presented.

Reviewer #1: Partly

Arthur’s reaction: Dear reviewer, I respect your comment on the technical soundness and the relationship between the data and the conclusion. Just to say few words in this regard, the experiment was done on 300 genotypes in alpha-lattice design replicated two times per testing site for two years. The experimental set up yielded a data of 12 replicates per genotype considering each replicated experiment across testing locations and years as a single environment. That is: 2 replication/location X 3 testing locations X 2 years = 12 replicates. The data collection (evaluation on level of resistance for each genotype) was done for a total of 3 times in 7 days interval starting from the date the spreader rows attained 50S (50% SEV and Fully Susceptible response (numerically S = 1.0)) Field Score. This has provided us 36 replicates of disease response data per genotype in the whole experiment. Hence, all the accompanied conclusions were drawn based on the analysis done at single environment level and combined over environments as well. Moreover, after your constructive suggestion under “5. Review Comments to the Author: no. (3)”, I have discarded the data of CHD-15 and KUL-15 from all subsequent analyses (because they don’t provide sufficient information). So, I believe this comment has improved the technical soundness of the manuscript as you may judge it from the revised submission.

Reviewer #2: Partly

Arthur’s reaction: Dear reviewer, I respect your comment on the technical soundness and the relationship between the data and the conclusion. Just to say few words in this regard, the experiment was done on 300 genotypes in alpha-lattice design replicated two times per testing site for two years. The experimental set up yielded a data of 12 replicates per genotype considering each replicated experiment across testing locations and years as a single environment. That is: 2 replication/location X 3 testing locations X 2 years = 12 replicates. The data collection (evaluation on level of resistance for each genotype) was done for a total of 3 times in 7 days interval starting from the date the spreader rows attained 50S (50% SEV and Fully Susceptible response (numerically S = 1.0)) Field Score. This has provided us 36 replicates of disease response data per genotype in the whole experiment. Hence, all the accompanied conclusions were drawn based on the analysis done at single environment level and combined over environments as well. Moreover, after the constructive suggestion from Reviewer #1 under “5. Review Comments to the Author: no. (3)”, I have discarded the data of CHD-15 and KUL-15 from all subsequent analyses (because they don’t provide sufficient information). So, I believe this comment has improved the technical soundness of the manuscript as you may judge it from the revised submission.

2. Has the statistical analysis been performed appropriately and rigorously?

Reviewer #1: N/A

Reviewer #2: Yes

Arthur’s reaction: thanks for the comment

3. Have the authors made all data underlying the findings in their manuscript fully available?

The [PLOS Data policy](http://www.plosone.org/static/policies.action#sharing) requires authors to make all data underlying the findings described in their manuscript fully available without restriction, with rare exception (please refer to the Data Availability Statement in the manuscript PDF file). The data should be provided as part of the manuscript or its supporting information or deposited to a public repository. For example, in addition to summary statistics, the data points behind means, medians and variance measures should be available. If there are restrictions on publicly sharing data—e.g. participant privacy or use of data from a third party—those must be specified.

Reviewer #1: No

Arthur’s reaction: Thanks for your comment. The data availability of the study has clearly been declared under “Data Availability Statement in the manuscript PDF file”. Unless I missed something unknowingly, I think the results of the study have been presented in the respective relevant parts of the manuscript. As you said however, I should have included the data behind the results particularly the reaction of each genotype (Severity (SEV), Field Response (RES) and Coefficient of Infection (CI) which strengthens the usability of the information. Accordingly, I have updated Supporting information S2 Table with disease reaction data of each genotypes across environments.

Reviewer #2: Yes

Arthur’s reaction: thanks for the comment

4. Is the manuscript presented in an intelligible fashion and written in standard English?

PLOS ONE does not copyedit accepted manuscripts, so the language in submitted articles must be clear, correct, and unambiguous. Any typographical or grammatical errors should be corrected at revision, so please note any specific errors here.

Reviewer #1: Yes

Arthur’s reaction: thanks for the comment

Reviewer #2: Yes

Arthur’s reaction: thanks for the comment

5. Review Comments to the Author

Please use the space provided to explain your answers to the questions above. You may also include additional comments for the author, including concerns about dual publication, research ethics, or publication ethics. (Please upload your review as an attachment if it exceeds 20,000 characters)

Reviewer #1: This work conducted a genome wide association study of yellow rust resistance on 300 Ethiopian durum wheat accessions. The results are helpful to resistance breeding. My suggestions:

(1) please show the phenotype data of spreader to judge the quality of field resistance evaluation.

Arthur’s reaction: Thanks for the comment. The spreader row, as detailed in the materials and methods section, is a mixture of 3 durum wheat (LD-357, Arendato and Local Red) and a bread wheat (Morocco) variety known for their relatively high susceptibility to Pst). They are mixed in the ratio of 1:1:1:1 and sown 20 cm from the experimental plots. The scoring or disease reaction was started when disease severity (SEV) is ≥ 50% and a field response (RES) of value 1.0 which is designated by “S” is observed on the spreader rows. A combination of the two values is usually recorded as “50S” during scoring. Besides, the level of infection on some of the known susceptible genotypes was also monitored to start evaluation. The reaction data of Local Red (one of the spreaders’ mix) and 4 other landraces (consistently susceptible across four of the environments) have been included in the revised manuscript as additional result table (Table 1). The level of infection on these five susceptible genotypes was assessed for it’s consistency of susceptibility and used as a basis to judge the suitability of the disease reaction data across environments for downstream analysis. You may see that in the revised manuscript with Track Changes. (Line: 196 – 198; Line:210 – 214 and in Table 1)

(2) please adding the phenotype data of analysed wheat lines in Table S1, which is important information for further genetics and breeding studies.

Arthur’s reaction: Thanks for the comment. The averaged data of Severity (SEV), Field Response (RES) and Coefficient of Infection (CI) for the analysed 293 wheat genotypes have been provided by updating supporting information S1 Table and now the name is S2 Table in the revised submission.

(3) For SEV, 100% of the accessions were classified as resistant (0≤SEV≤10) at CHD_15 and 99.7% at KUL_15 . For RES, 96.6% of the accessions fell under resistant class at CHD_15 and 97.63% at KUL_15. The low Pst infestation cannot provide useful information in following analysis but makes the analysis complex and confusion. I suggested to discard the data of the two experiments in further analysis.

Arthur’s reaction: Thanks for the comment. You are absolutely right about that; I already felt that during the analysis and interpretation of the results and I even run a separate analysis using data of the four environments (CHD_16, KUL_16, MER_15 and MER_16) excluding the two you suggested (CHD_15, KUL_15). This is a very valuable suggestion and I have updated all parts of the manuscript related to this change and you may have a look to the revised manuscript with Track Changes.


Other a few corrections in the attached file.

Arthur’s reaction: Thanks for the comment. I have located the place and made the amendment accordingly in the revised manuscript with Track Changes.

Reviewer #2: This manuscript reported genome-wide association mapping of yellow rust resistance locus in large number of Ethiopian Durum Wheat germplasms. The author identified 12 SNPs significantly associated with yellow rust resistance across four chromosomes, meanwhile this provides SNPs for tracking the QTL associated with yellow rust resistance in durum wheat improvement programs. However, several revision should be done before the manuscript can be accepted for publication.

1.After the introduction of Yr genes identification, please add some description of Yr genes that have been identified in Durum wheat in detail.

Arthur’s reaction: thanks for the comment. I have located the place in the document and made the amendment in the revised manuscript with Track Changes: (Line:99 – 106 and S1 Table)

2.Please added some introduction of MAS of the Yr genes in Introduction.

Arthur’s reaction: thanks for the comment. I have located the place in the document and made the amendment in the revised manuscript with Track Changes: (Line:106 - 110)

3.Introduction part is too long. Please delete some unessential description.

Arthur’s reaction: Thanks for the comment. I have trimmed out some of the statements I think are unessential and you may check this from the revised manuscript with Track Changes (Line: 79 – 82; Line:87 – 92; Line:120 – 122; Line:134 – 145).

4.If the germplasm resources in this study can be provided to the public, and where we can get?

Arthur’s reaction: thanks for the comment. It is found at the National Agricultural Biotechnology Research Centre, wheat biotechnology research program in Ethiopia. Access for research purpose only can be provided through officially signed MTA (Material Transfer Agreement) with the consent of EBI (Ethiopian Biodiversity Institute) via a collaborative research project (joint venture) with EIAR-NABRC.

5.In line 269, the number “829100000” should be changed in to “829,100,000”

Arthur’s reaction: Thanks for the comment. I have located the place and made the correction in the revised manuscript with Track Changes (Now is located in Line: 297).

6.The Tables in this study should be changed into the “Three-line table” format.

Arthur’s reaction: thanks for the comment. Under <https://journals.plos.org/plosone/s/tables> and PLOSOne_formatting_sample_main_body.pdf, it says that “Tables must be cell based in Microsoft Word or embedded with Microsoft Excel”. I was strictly following the “Submission Guidelines” and that is why the tables were cell-based. But now, to comply with the constructive comment, all the tables in the manuscript have been presented in the three-line-table format.

7.If the elite resistant loci identified in this study have been transferred into susceptible cultivars in the production? If yes, please state it in Discussion

Arthur’s reaction: Thanks for the comment. As the scope of the current study did not include this objective, we did not transfer it into any susceptible cultivar. However, that is a nice reminder and is already indicated as a future direction in the conclusion part.

8.The References have Non-uniform format, please revised them meticulously.

Arthur’s reaction: thanks for the comment. As much as I can, I have addressed this.

6. PLOS authors have the option to publish the peer review history of their article ([what does this mean?](https://journals.plos.org/plosone/s/editorial-and-peer-review-process#loc-peer-review-history)). If published, this will include your full peer review and any attached files.


If you choose “no”, your identity will remain anonymous but your review may still be made public.


**Do you want your identity to be public for this peer review?** For information about this choice, including consent withdrawal, please see our [Privacy Policy](https://www.plos.org/privacy-policy).

Reviewer #1: No

Reviewer #2: **Yes:** Pengtao Ma

[NOTE: If reviewer comments were submitted as an attachment file, they will be attached to this email and accessible via the submission site. Please log into your account, locate the manuscript record, and check for the action link "View Attachments". If this link does not appear, there are no attachment files.]

While revising your submission, please upload your figure files to the Preflight Analysis and Conversion Engine (PACE) digital diagnostic tool, <https://pacev2.apexcovantage.com/>. PACE helps ensure that figures meet PLOS requirements. To use PACE, you must first register as a user. Registration is free. Then, login and navigate to the UPLOAD tab, where you will find detailed instructions on how to use the tool. If you encounter any issues or have any questions when using PACE, please email PLOS at [figures@plos.org](mailto:figures@plos.org). Please note that Supporting Information files do not need this step.

*In compliance with data protection regulations, you may request that we remove your personal registration details at any time.* [*(Remove my information/details)*](https://www.editorialmanager.com/pone/login.asp?a=r)*. Please contact the publication office if you have any questions.*
